# The Role of Preoperative Onyx Embolization by Transnasal Direct Puncture in the Treatment of an Advanced Juvenile Nasopharyngeal Angiofibroma

**DOI:** 10.7759/cureus.40984

**Published:** 2023-06-26

**Authors:** Tiago Hilton Vieira Madeira, Augusto Meneghelli Galvão Gonçalves, Amanda Viguini Tolentino Correa, Clauder Oliveira Ramalho, Alexandre Nascimento Ottoni

**Affiliations:** 1 Neurosurgery and Interventional Neuroradiology, Hospital Santa Rita de Cássia, Vitória, BRA; 2 Interventional Neuroradiology, Hospital Santa Rita de Cássia, Vitória, BRA; 3 Medical School, Faculdade Brasileira Multivix, Vitória, BRA; 4 Neurosurgery, Hospital Santa Rita de Cássia, Vitória, BRA

**Keywords:** head and neck tumor, direct puncture embolization, angiofibroma, onyx copolymer, preoperative care

## Abstract

Juvenile nasopharyngeal angiofibroma (JNA) is a rare and locally aggressive tumor that commonly presents with painless nasal obstruction and severe, recurrent epistaxis. In this case report, we describe the successful management of a Radkowski stage IIIA JNA with extensive arterial supply from the internal carotid artery (ICA). Transnasal direct puncture embolization using Onyx (Medtronic, Minneapolis, Minnesota) was employed to effectively devascularize the tumor, enabling radical surgical resection in a single piece via endonasal and transmaxillary endoscopic approaches. The patient did not require blood transfusion and was discharged without neurological impairment. The effectiveness of preoperative embolization as a treatment strategy for JNA is also discussed.

## Introduction

Juvenile nasopharyngeal angiofibroma (JNA) is a rare vascular tumor that represents a small percentage of all head and neck tumors [1]. For some authors, it usually originates from the sphenopalatine foramen, which is formed by the sphenoid process of the palatine bone, the horizontal ala of the vomer, and the root of the pterygoid process [2]. However, the posterior part of the pterygopalatine fossa is also cited in the literature as its site of origin [3]. Despite its benign nature, it can exhibit locally aggressive behaviors, including extra- and intracranial invasion by eroding the skeletal boundaries and spreading through anatomical openings. JNAs usually expand first into the nasopharynx and nasal cavities, but it may also affect the maxillary sinus, pterygoid region, infratemporal fossa, and orbit [3,4]. Intracranial extension is a late event occuring in up to 36% of patients, commonly through the extradural middle cranial fossa, but it may also spread to the anterior cranial fossa, pituitary fossa, and cavernous sinus [3-6].

Histological analysis reveals numerous vascular channels that are typically lined with a single layer of endothelium and lack a complete muscular layer, which contributes to the tumor’s propensity for massive hemorrhage [2]. Nasal obstruction and recurrent unilateral epistaxis are the most common initial symptoms [4,7].

Surgical resection is the most effective treatment for this condition [7]. However, the tumor’s tendency to extend into the intracranial space and erode the skull base, combined with its high vascularization, can pose challenges for the surgeon due to the potential for profuse intraoperative bleeding and attachments [5].

Preoperative embolization can help prevent extensive intraoperative blood loss (IBL), particularly in patients with intracranial invasion. When the tumor’s arterial supply comes from the external carotid artery (ECA), transarterial embolization with polyvinyl alcohol (PVA) microparticles or microspheres is the preferred technique. In cases where the tumor has an extensive supply from the internal carotid artery (ICA) branches, endovascular embolization carries a high risk of ischemic complications due to the reflux of microparticles or ethylene-vinyl alcohol copolymer (EVOH). In these situations, direct tumor puncture followed by embolization using EVOH or acrylic glue would presumably be an excellent alternative for promoting total or near-total tumor devascularization [5,7-11].

## Case presentation

A 28-year-old male presented with a progressive protrusion of his right eye over a period of six months. His medical history includes a previous surgical treatment of JNA restricted to the nasal cavity four years prior. During the procedure, he underwent preoperative embolization of the internal maxillary artery (IMAX) using PVA microparticles. Despite the embolization, it was not possible to fully remove the lesion due to excessive IBL.

**Figure 1:**
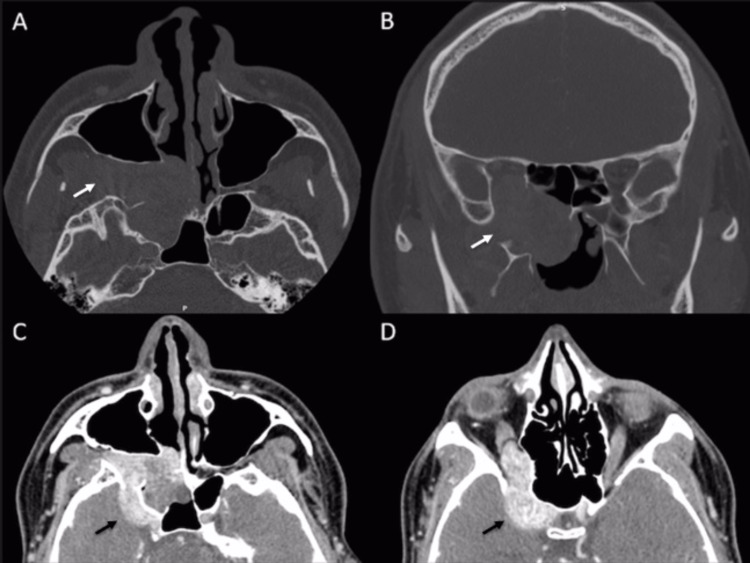
Preoperative sinonasal CT scan The arrows in 1A, 1B, 1C, and 1D depict a lobulated hypervascular lesion with remodeled adjacent bone structures, an enlarged sphenopalatine fissure and pterygomandibular fossa, and an enlarged right foramen rotundum and inferior orbital fissure with extension to the posterior regions of the right orbital fossa. This lesion extends medially and inferiorly toward the nasal fossa. CT: computed tomography

**Figure 2:**
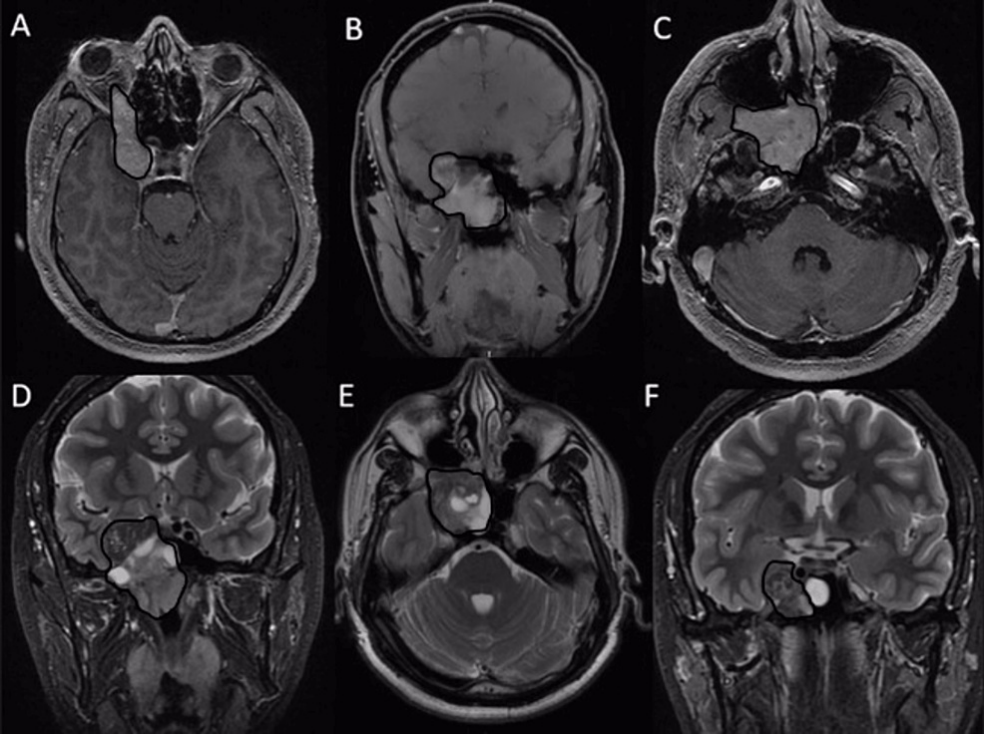
Preoperative brain MRI The nonencapsulated lobulated hypervascular lesion depicted in images 2A, 2B, and 2C can be observed extending from the right orbital fossa anteriorly to the right temporal fossa posteriorly and occupying the right cavernous sinus. In images 2D, 2E, and 2F, the characteristic MRI appearance of this entity is evident, with areas exhibiting "flow voids" (commonly referred to as "salt and pepper") [12] and some cystic foci visible in T2-weighted hyperintense images. The epicenter of this tumor is located in the right cavernous sinus, and it extends medially to the right sphenoid sinus and laterally to the middle cerebral fossa. MRI: magnetic resonance imaging

Sinonasal computed tomography (CT) and magnetic resonance imaging (MRI) (Figures 1, 2) showed the presence of a large tumor in the posterior nasal cavity spreading to the right side of the pterygopalatine fossa, infratemporal fossa, maxillary sinus, orbit, and cavernous sinus.

**Figure 3:**
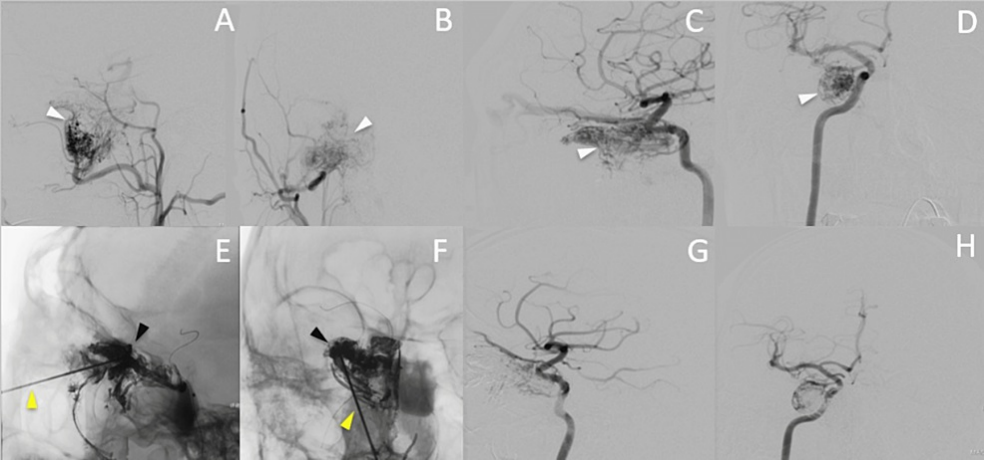
DSA of tumor vascularization The right ECA is displayed in lateral (3A) and anteroposterior (3B) projections, demonstrating tumoral vascularization. Following IMAX embolization, the right ICA is depicted in lateral (3C) and anteroposterior (3D) projections, exhibiting a significant residual blush. DPT Onyx embolization is displayed in lateral (3E) and anteroposterior (3F) projections. Final control images of the right ICA in lateral (3G) and anteroposterior (3H) projections reveal nearly complete devascularization of the tumor. White (3A-D), black (3E-F) and yellow (3E-F) arrows indicate, respectively, the tumor’s hypervascular blush, the onyx cast and the needle. DSA: digital subtraction angiography; ECA: external carotid artery; IMAX: internal maxillary artery; ICA: internal carotid artery; DPT: direct puncture technique

The digital subtraction angiography (DSA) examination revealed a highly vascularized tumor, primarily supplied by the IMAX branches, ethmoidal branches from the ophthalmic artery, and dural branches of the inferolateral trunk (cavernous segment of ICA) (Figures 3A, 3B). A Rebar 27 microcatheter (Medtronic, Minneapolis, Minnesota) (proximal outer diameter = 2.8f, inner diameter = 0.027”, total length = 150 cm) guided by a Traxcess 14 microwire (Microvention/Terumo, Tustin, California) was successfully navigated to the IMAX for the purpose of embolization using Embozene (Boston Scientific, Marlborough, Massachusetts) in sizes of 100, 400, and 500 mm. Immediate DSA control showed significant reduction of the residual tumoral blush from the ICA branches (ethmoidal arteries and inferolateral trunk) (Figures 3C, 3D).

To address the tumor, we implemented a technique known as direct intratumoral embolization using Onyx (as depicted in Figures 3E, 3F). The procedure involved inserting a 22-gauge BD spinal needle (Becton Dickinson, New Jersey) through the patient's right nostril, using fluoroscopic guidance to position the needle within the tumor bed. To prevent blood reflux, a stopcock was attached to the needle. The needle's dead space was then filled with dimethyl sulfoxide (DMSO), and the Onyx embolic liquid was injected. To prevent migration of the Onyx to intracranial vessels, a Hyperglide balloon 4x20 mm (Covidien/eV3, Irvine, California) was inflated at the cavernous and ophthalmic segment of the ICA during the injection. In addition, two additional needles were inserted at different locations within the lesion, and five vials of the aforementioned embolic agent were injected (1.5 mL each vial). Final DSA control showed near-total devascularization of the lesion (as shown in Figures 3G, 3H). It is worth noting that a crucial aspect of this technique is to close the stopcock immediately after removing the DMSO syringe to prevent blood backflow, which could wash out the DMSO and cause early precipitation of the Onyx within the needle.

**Figure 4:**
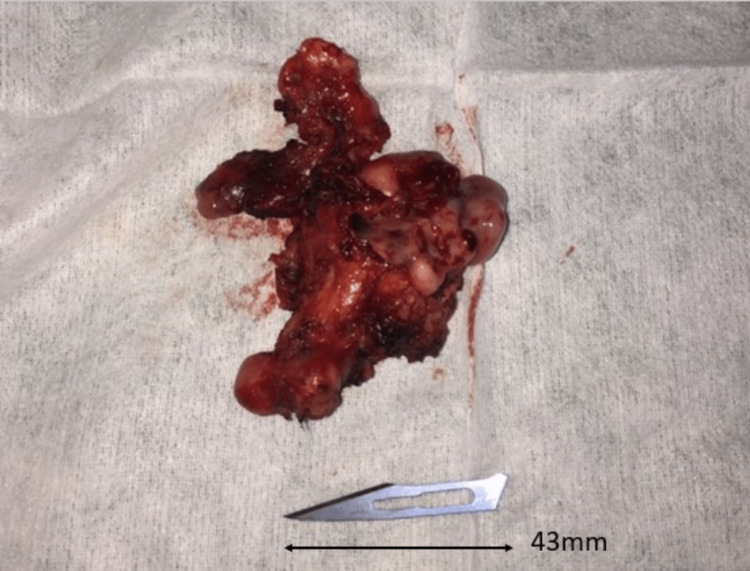
Tumor after surgical en bloc resection A 43 mm scalpel blade, number 11, is placed next to the tumor for the purpose of comparative sizing.

**Figure 5:**
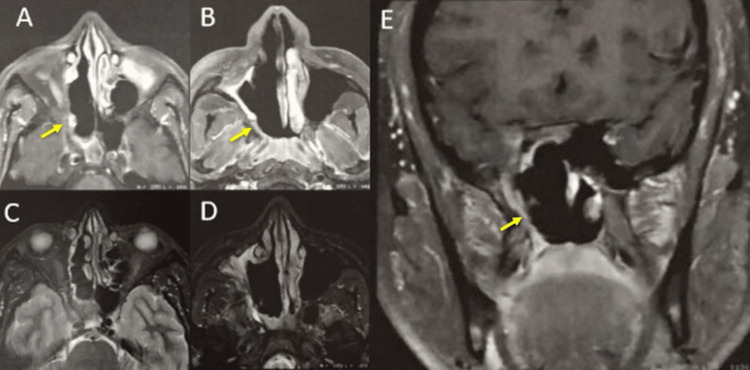
MRI following surgical intervention A partial right ethmoidectomy and right turbinectomy were performed, including the surgical excision of a cavity connecting the nasal fossa and right ethmoid cells as well as the right maxillary sinus. Upon examination, we observed an enlarged right choana, as depicted by the yellow arrows in images 5A, 5B, and 5E. T2-weighted axial images 5C and 5D strongly imply a gross total resection of the tumor. MRI: magnetic resonance imaging

Following the embolization, the patient underwent endoscopic surgery (combined endonasal and sublabial transmaxillary approach) for the en bloc removal of the tumor (as depicted in Figure 4). Blood transfusion was not necessary. The recorded preoperative and immediate postoperative hemoglobin levels were 14.31 and 11.65 mg/dl, respectively. Post-operative images indicated a gross total resection of the tumor (as shown in Figure 5).

## Discussion

JNAs are primarily supplied with blood by the ECA through the internal maxillary or ascending pharyngeal arteries. As the tumor grows, it may also acquire blood supply from adjacent structures, such as branches of the ipsilateral and occasionally contralateral ICA and the contralateral external carotid system [8]. In fact, up to 36% of JNAs have been found to have bilateral ECA supply on angiography. The extent of bilateral vascularization and ICA tumor feeding can impact the IBL and overall outcome [5].

Preoperative embolization is a procedure that reduces the length of the operation and morbidity by decreasing the potential for IBL and providing more safety during the excision of the tumor. The transarterial approach, which involves accessing the tumor through the arteries, is the most commonly used technique and reduces both blood loss and operation time [13]. However, this method can be time-consuming due to the need to catheterize multiple feeding arteries, and it may be ineffective in cases where the vessels are small, have unfavorable angles of origin, or are tortuous.In addition, the risk of accidental embolization into the cerebral circulation is increased when extracranial-to-intracranial anastomoses are presented [8,13]. Once the tumor has extended to the skull base, the involvement of the vertebral artery and ICA arterial feeders can make transarterial embolization more dangerous and complex [13].

Direct puncture embolization (DPE) provides a direct and efficient way to access the tumor vasculature. An intratumoral angiogram is used to evaluate the vascular structure of the tumor, arterial reflux, venous drainage, and risk of extravasation. Injections are performed under blank roadmap guidance and may include the use of a non-detachable balloon placed across the origin of the branches of the ICA to prevent accidental embolization. DPE allows greater filling of the vascular compartment and creates a casting of the tumor vessels, resulting in more efficient devascularization than transarterial embolization and improved surgical outcomes [13].

Elhammady et al. compared 10 patients with JNAs who underwent preoperative embolization by a transarterial (n = 5) versus DPE (n = 5) using Onyx. Significant intraparenchymal penetration of the embolic material was possible in all DPE tumors, and none of the tumors were embolized by the transarterial route. The mean percent tumor devascularization in the transarterial group was 77% compared with 93% at the direct intratumoral embolization group [8].

Gao et al. reviewed 50 consecutive JNA resections at 43 patients - 39 cases underwent embolization by the transarterial particulate technique and 11 by direct intratumoral embolization using Onyx. The mean IBL for the Onyx group was 569.1 ± 700.1 mL, and the mean IBL for the particulate group was 1348.7 ± 932.2 mL (one-tailed Student’s t-test, p = 0.016). The mean units of packed red blood cells (PRBCs) used in the Onyx group were 0.45 ± 1.0 units, and the mean PRBCs used in the particulate cohort were 1.56 ± 2.01 units (p = 0.008). The improved surgical outcome, with less IBL, less PRBC transfusion during the surgery, less operative room time, and shorter total hospital stay, would suggest that Onyx provides greater penetration at the capillary bed of the tumor and better outcome [9].

Gemmete et al. compared the results of preoperative devascularization of JNA in 39 patients treated with particulate material versus nine patients treated with DPE using only EVOH (Onyx). The mean estimated blood loss (EBL) for the Onyx group was 567.7 mL, and the mean EBL for the particulate group was 1258.6 mL (one-tailed Student’s t-test, p= 0.043). The mean unit of PRBCs used in the Onyx group was 0.29 ± 0.76 U (p = 0.003), and the mean PBRC used in the particulate cohort was 1.56 ± 2.01 U [4].

The Onyx polymer, when in contact with blood, initially precipitates at the peripheral portion of the blood vessel, and this particular feature makes its choice preferred over N-butyl-2-cyanoacrylate (NBCA), which polymerizes fast with blood contact. This peculiarity enables a longer controlled injection with better penetration of the vascular bed compared to NBCA and allows the surgeon to stop the lesion injection once the Onyx starts to enter an arterial pedicle, a venous outflow vessel, or a dangerous ECA/ICA anastomosis. After this portion of the lesion solidifies, the injection can be restarted. Then, the Onyx fills another portion of the lesion, following the path of least resistance [9].

The quality of life (QOL) in patients with benign and malignant sinonasal tumors after surgery was assessed through patient-reported outcome measures (PROMs) at the study of Chow et al. The authors highlight the scarce data on this topic and the need for further long-term studies and point that the endoscopic approach was associated with improved QOL within several months after surgery [14].

We reported a case of a Radkowski stage IIIA JNA with an extensive arterial supply from ICA. The DPE technique using Onyx devascularized most of the residual blush from ICA branches. It allowed a radical surgical resection of the lesion in a single piece, including intraorbital and cavernous sinus portions, exclusively by the endonasal and transmaxillary endoscopic approach, without significant bleeding. No blood transfusion was needed, and the patient was discharged without neurological impairment.

## Conclusions

The authors present a case of an advanced JNA managed successfully through surgical resection. Preoperative direct intratumoral embolization using the EVOH copolymer, specifically Onyx, has been shown to significantly contribute to the successful treatment of JNA. Its slower precipitation properties provide adequate control during the injection of the embolic agent, enabling the performance of a safe and complete resection of a Radkowski stage IIIA JNA through an exclusively endoscopic approach with minimal IBL and no neurological impairment.
